# Diet and Spatial Ecology Influence Red-Legged Partridge Exposure to Pesticides Used as Seed Treatment

**DOI:** 10.1021/acs.est.3c03905

**Published:** 2023-09-25

**Authors:** Elena Fernández-Vizcaíno, François Mougeot, Xabier Cabodevilla, Mario Fernández-Tizón, Rafael Mateo, María J. Madeira, Manuel E. Ortiz-Santaliestra

**Affiliations:** † Instituto de Investigación en Recursos Cinegéticos (IREC) CSIC-UCLM-JCCM, Ronda de Toledo 12, Ciudad Real 13005, Spain; ‡ Conservation Biology Group, Landscape Dynamics and Biodiversity Program, Forest Science and Technology Centre of Catalonia (CTFC), km 2, Solsona 25280, Spain; § Terrestrial Ecology Group (TEG-UAM), Department of Ecology, Universidad Autónoma de Madrid, Calle Darwin 2, Madrid 28049, Spain; ∥ Department of Zoology and Animal Cell Biology, Faculty of Pharmacy, University of the Basque Country (UPV/EHU), Paseo de la Universidad 7, Vitoria-Gasteiz 01006, Alava, Spain

**Keywords:** treated seeds, pesticide exposure, farmland birds, diet, spatial ecology

## Abstract

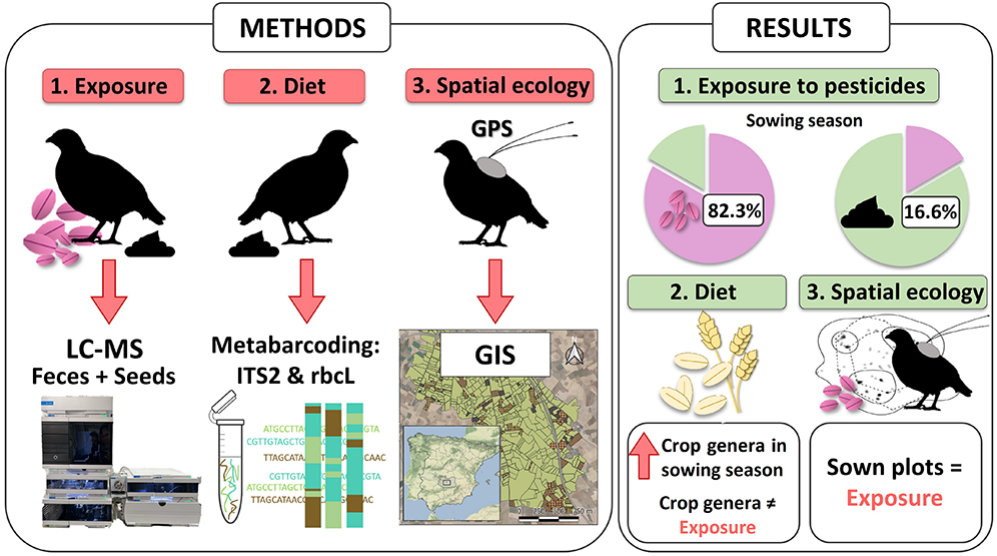

Seed treatment with pesticides is an extended agricultural practice with a high risk to granivorous birds that consume those seeds. To characterize that risk, it is necessary to understand the ecological factors that determine the exposure chances of birds to treated seeds. We investigated how pesticide uptake by red-legged partridges was related to cultivated plant ingestion and to the use of recently sown fields. We analyzed pesticide residues in 144 fecal samples from 32 flocks and determined the plant diet composition using DNA metabarcoding. Habitat use was studied through the monitoring of 15 GPS-tagged partridges. We confirmed, through the analysis of seeds, that >80% of cereal fields from the area had seeds treated with triazole fungicides. Tebuconazole was detected in 16.6% of partridges’ feces. During the sowing season, cultivated plants accounted for half of the plant diet, but no association was found between cultivated plant consumption and pesticide intake. GPS tracking revealed that tebuconazole was detected in feces when partridges had recently used sown fields, whereas nonexposed partridges showed no overlap with recently sown areas. Our results highlight the need to incorporate field ecology into the characterization of pesticide exposure to improve the efficacy of environmental risk assessment.

## Abstract 2

We determined the role of diet and spatial behavior as drivers of exposure to pesticide-treated seeds in granivorous birds.

## Introduction

The treatment of seeds with pesticides is an extended agricultural practice that consists of coating the seeds with a pesticide before sowing. Treated seeds may not be properly buried or spilled during sowing, which makes them accessible for granivorous birds as a food source. The exploitation of this resource is stimulated in winter cereal crops (e.g., wheat, barley, rye, and oats) in the Mediterranean region because their sowing seasons coincide with a period (i.e., autumn and winter) of scarcity of natural food sources. The consumption of treated seeds represents a very specific risk for granivorous farmland birds,^1^ such as the red-legged partridge, whose diet includes a large proportion of cultivated winter cereal seeds.^2–4^ Consequently, this species may be attracted to arable fields during the cereal sowing season.^3,5–7^ In Spain, red-legged partridge populations have declined by 40% since 1998.^8^ In central Spain (Castilla-La Mancha region), its populations were reduced by 51% during 2010–2017,^9^ and the declines have been linked to agricultural intensification.^9,10^

Triazole fungicides are widely used for cereal seed treatment in current agriculture. Over the past decade, a growing number of studies have revealed the adverse effects that these fungicides used for seed treatment have on granivorous birds, with the potential to alter the synthesis and regulation of steroids, including sexual hormones, and reduce the reproductive capacity of birds.^11–13^ The exposure of birds to triazole fungicides through the consumption of treated seeds has been confirmed in the wild. Several studies in Spain have shown detectable levels of eight pesticides (including five triazoles) in ca. 30% of digestive contents from hunted red-legged partridges,^2,3^ and Fernández-Vizcaíno et al.^14^ recently showed that 18.6% of fecal samples from wild partridges contained triazole fungicides used for seed treatment in central Spain.

Despite the availability of studies investigating the toxicity of pesticides used as seed treatments and reports on the levels of exposure to these pesticides in wild birds, there is still limited knowledge of the ecological factors that modulate exposure in nature. For example, the diet of birds living in agricultural environments is expected to play a major role in determining the risk of exposure to pesticides, as shown by studies reporting how a greater consumption of cereal sown seeds is associated with a higher prevalence of triazole fungicides in the digestive contents of red-legged partridges.^2,3^ Moreover, the dietary exposure of birds to pesticides has been found to be associated with landscape characteristics, with a reduction of pesticide uptake in areas with heterogeneous landscapes and increased availability of noncultivated food sources to birds.^2^ Agricultural landscape structure can influence the spatial ecology of animals, and feeding habits are one of the most important drivers of this association between landscape and spatial behavior.^15–17^ This association implies that the exposure of farmland wildlife to pesticides would be determined by their habitat use.^18^ For the specific case of seed treatments, the availability of sown seeds on field surfaces was shown to be a key determinant of pesticide exposure;^19–22^ hence, we can hypothesize that the relative use of those fields by birds will be a major driver determining their exposure chances.

To complete an appropriate exposure characterization to support the environmental risk assessment of pesticides used as seed treatments, it is necessary to further understand the factors that modulate the uptake of pesticide-treated seeds by birds. For this purpose, we designed a field-based study to test the hypothesis that exposure of red-legged partridges to pesticides used as seed treatment is associated with (i) the extension and temporal distribution of treated seed use, (ii) the frequency of ingestion of cultivated plants (during the sowing season, cultivated plants are mostly available as seeds), and (iii) the use of recently sown fields by birds. For this purpose, we studied the diet, habitat use, and pesticide exposure of wild red-legged partridges in central Spain during the sowing season in an agricultural landscape dominated by winter cereal crops. We mapped crop types, monitored the timing of cereal sowing, and collected samples of sown seeds to identify the occurrence and type of pesticide treatments used in the study area. Simultaneously, we tagged 15 red-legged partridges with high-resolution GPS-tracking devices to monitor their spatial ecology and use of cropped fields during the sowing season. We collected feces from tagged birds and other partridges from the same flocks to study diet composition using metabarcoding techniques and to determine pesticide exposure through the analysis of pesticide residues in the excreta.

## Materials and Methods

### Study Area and Land Use

The study was conducted in Miguelturra, Castilla-La Mancha, central Spain (38°57′53″N and 3°53′28″W), a main population stronghold for the red-legged partridge in Spain and Europe (Figure S1).^8,10^ The study area is a typical agricultural area of the Spanish southern Plateau (mean elevation of 635 m above sea level) dominated by dry cereal fields interspersed with vineyards and olive groves (Corine, 2018). The sampled area comprised 522 agricultural plots (fields) with a total extension of 1.39 km^2^ (1393.25 ha) and was monitored between September 2017 and May 2018 to map crop types and determine the timing of sowing. From September to December, during sowing, agricultural plots were sampled weekly to record land use and, in the case of herbaceous crop plots, to determine the timing of sowing. Similar samplings were conducted between January and May but were performed monthly instead of weekly. Of the 522 agricultural plots in the area, 9.0% were cultivated with olive groves, 5.0% with vineyards, 3.3% with fruit orchards, and 6.5% were anthropized areas (e.g., buildings, gardens, and parking lots). The remaining plots (76.2%) were used to cultivate herbaceous crops, although not all plots were sown every year due to crop rotation, including fallows. Considering the surface area, 5.61% (78.11 ha) was occupied by olive groves, 6.06% (84.42 ha) by vineyards, 2.67% (37.14 ha) by fruit orchards, 2.38% (33.17 ha) by anthropized areas, and 83.29% (1160.38 ha) by herbaceous crops.

Out of the 414 plots eventually used for herbaceous crops, 216 were sown during the study season, comprising a total extension of 650.95 ha (46.72% of the area). In order to characterize the extension and type of seed treatments used in the area, we collected seed samples from 101 of those plots (Figure S2), which we used to identify the specific pesticide treatments used by farmers in the area. Although treated seeds can be easily recognized due to the colorants applied by dyes added to commercial formulations, we collected both colored and uncolored seeds. Seed samples from different plots were collected at different times relative to the sowing date so it is very likely that the pesticide degradation level varied among seed sample collections. Therefore, we characterized seed treatment using pesticide prevalence, irrespective of the measured concentrations.

Sown seed persistence on the field surface depends on weather conditions (e.g., rainfall) and could not be monitored in all plots. For our analyses, we considered that cereal fields would have available seeds for 15 days after sowing. This period is consistent with the average times of permanence of sown seeds on the field surface reported by Lopez-Antia et al.^3^ (11 days) and Lennon et al.^19^ (14 days), but shorter than the maximum permanence time reported by Lopez-Antia et al.^3^ (25 days). Data collected during our study indicated that seeds can remain available on the field surface for up to 6 weeks after sowing, but we considered that the 15 day period was a conservative estimate of seed persistence and availability to birds.

### Study Species and Feces Collection

To study the habitat use of partridges during the sowing season, we captured one adult red-legged partridge per flock (*n* = 15) to fit them with high-resolution GPS-tracking devices. In September–October of 2017, we located and captured birds at night using a thermal camera, a spotlight, and a large hand-held net.^23^ We fitted captured red-legged partridges with a backpack GPS radio transmitter (Ecotone model CREX 12g, Poland) that recorded bird locations with a 5m resolution up to eight times per day. Recorded positions were sent via the GSM network to the device’s online application. Handling time was below 20 min and transmitter weight represented 2.2–3.3% of bird weight, which is below the maximum 5% of body mass recommended for GPS emitters.^24^ To study pesticide exposure and plant diet composition, we collected fecal samples from roosting partridges between September 2017 and May 2018. With this purpose, partridge flocks were located at night by using the same method as that used for deploying GPS transmitters. As we approached the birds, they flew away, and we collected the feces exactly from the spots where the animals had been roosting. This procedure allowed us to ensure that the feces had been deposited during that night; some of the feces were probably deposited when birds flew away as this is a common response of partridges when escaping from predators.^25^ When partridges were in flocks, we collected feces that were at least 2 m away from each other to minimize that samples belonged to the same individual within the flock. We collected a total of 144 fecal samples (Figure S2). Eight samples were collected from 4 flocks in late summer (September, before sowing), 84 samples were collected from 15 different flocks in autumn (October–December, during peak sowing season), 33 samples were collected from 5 flocks in winter (January–February, when some seeds from late sown plots could still be available), and 19 samples were collected from 8 flocks in spring (March–May, when all herbaceous crops had germinated). All samples were collected with clean forceps, stored in individual zip bags with reference to the date of collection, agricultural plot, and flock identity, and kept at −80 °C until analysis.

### Pesticide Residue Analysis

The presence of pesticides was determined using liquid chromatography coupled to single quadrupole mass spectrometry with electrospray ionization (LC-ESI-MS) using an Agilent chromatograph −1100 series and Quadrupole LC/MS—6110 with a multimode source. We analyzed feces and seed samples following the method described by Lopez-Antia et al.^26^ with modifications of the detection protocol indicated by Fernández-Vizcaíno et al.^2^ We screened a total of 15 active ingredients, some of which were already banned when the study took place but that have been historically used on cereal fields in the region, and a synergist (piperonyl butoxide) added to some fungicide formulations (Table S1). Details of the methodological procedures followed to analyze pesticide residues in feces and seeds are provided in the Supporting Information (Section 1.1).

### Diet

To describe the plant diet of red-legged partridges, we applied DNA-metabarcoding techniques to fecal samples, for which we amplified two gene regions: the internal transcribed spacer 2 (ITS2), a nuclear barcode, and the large-chain subunit of the ribulose-1,5-bisphosphate carboxylase/oxygenase (rbcL), a chloroplastic barcode. Details of the methodological procedures for the analysis of diet are provided in the Supporting Information (Section 1.2).

### Spatial Ecology

For the spatial ecology study, we considered only the flocks monitored during autumn (Figure S2). During that season, partridges are organized into multifamily groups, which form in late summer and persist throughout the autumn, remaining stable until the mating season in the late winter.^27–30^ Therefore, we assumed that the GPS positions shown by a given animal were indicative of where the entire flock had been. Under this assumption, we were able to establish an association between the plots used by the GPS-tagged animals and the diet and pesticide exposure measured in the fecal samples collected from the flocks to which they belonged. All recorded GPS positions were analyzed using QGIS (Buenos Aires, 2022). We established positive associations when a GPS position between 18:00 and 20:00 h of the day of feces collection (i.e., a GPS position indicative of the location of the animal shortly before feces collection where the birds slept) was within 200 m of the centroid of the plot where the feces were collected. Based on these premises, we were able to link GPS-tagged bird locations to fecal samples for six out of the 15 flocks that were sampled in autumn. Given that triazole fungicides, the type of pesticides most commonly used for seed treatment in our study area,^2^ can be detected in feces for up to 72 h postingestion,^31^ we mapped the home range of the monitored flocks using the GPS positions collected during the 3 days (72 h) prior to feces collection. We used QGIS to determine home ranges using minimum convex polygons (MCPs) and the GPS locations of the tagged birds that belonged to the sampled flock. We then determined if the home range (MCP) overlapped or not with a recently sown plot (i.e., plots that were sown within the last 15 days) and if pesticides were detected or not in the feces collected from the partridge flock.

In addition, in order to know if the spatial ecology of partridges could be used as a proxy to estimate the risk of exposure from the ingestion of pesticide-treated seeds, we characterized the habitats within three concentric circles of 121, 234, and 296 m of radius, whose center was the centroid of the agricultural plot where feces were collected (*n* = 15 flocks). The selected surface areas correspond to the minimum, mean, and maximum extension of the calculated 3 day MCPs of the monitored flocks. As with the MCPs of the GPS-tracked animals, we checked whether the areas within the circles overlapped or not with recently sown plots.

### Statistical Analysis

We used SPSS v.24 software for statistical analyses. The significance level of all analyses was set at *p* < 0.05. The normality of the dependent variables and covariates was checked using Kolmogorov-Smirnov tests. To determine differences among seasons in the consumption of cultivated plants and therefore in the potential risk of pesticide ingestion, we ran two generalized linear models (GzLM), one for each gene (ITS2 and rbcL), using as the relative read abundance (RRA) of plant genera corresponding to cultivated plants (sum of barley, wheat, oats, peas, and vetch) a dependent variable and with the season as a fixed factor. When significant differences between seasons were detected, we used the least significant difference for pairwise comparisons.

To focus on the risk associated with treated seed ingestion, subsequent analyses were conducted using only autumn data (sowing time). Because we expected that data on ingestion of the different plant types were associated, and to avoid collinearity issues, we conducted principal component analyses (PCA) for each of the two analyzed genes, including the six diet components (barley, wheat, oats, peas, vetch, and wild plants) as input variables. For subsequent analyses, we considered the principal components (PCs) with an eigenvalue >1, which resulted in four PCs for ITS2 data and three PCs for rbcL data.

We used two GzLMs (one per gene) with a binomial error distribution and a logit link function to analyze the influence of the autumn diet on pesticide exposure probability. The initial model included the presence or absence of pesticides in feces as a dependent variable and the diet’s PCs as covariates. For all GzLM analyses, the initial model included all covariates and we performed a backward selection procedure, removing nonsignificant terms from the initial models.

In order to know if the presence of pesticides in feces was related to the use of recently sown plots by flocks with GPS-tracked partridges, we built a contingency table with the presence/absence of pesticides in feces and the use (yes or no) of recently sown areas (within 3 days prior to feces collection) as table entries. We tested for the difference between expected and observed frequencies in table cells using chi-square tests. The same statistical procedure was used to determine whether the presence of recently sown plots inside the home range (calculated using data collected 3 days before sample collection) increased pesticide exposure (detection of pesticides in fecal samples) using data from 15 partridge flocks monitored during the autumn. In this case, three contingency tables were built to test the influence of the presence of recently sown plots around the location of sample collection, using three areas whose radius corresponded to the minimum, mean, and maximum extension of the calculated MCPs.

## Results and Discussion

### Exposure Assessment: Pesticide Residues Detected in Partridge Feces

Pesticides were detected in 15 of the 144 analyzed feces, corresponding to five out of the 32 sampled flocks. We detected only two active ingredients, which were triazole fungicides. Most positive samples were found in autumn, coinciding with the sowing season, and tebuconazole was the only compound detected in feces collected during that period (Figure 2). Overall, 14 of the 84 individual samples collected in autumn, corresponding to 4 of the 15 flocks, showed detectable levels of this fungicide. The other detected compound, difenoconazole, was found in a single fecal sample collected in spring (Table 1).

**Table 1:** Pesticide Exposure in Wild Red-Legged Partridge Flocks^a^

					pesticide concentration				
exposed flocks ID	feces collection month	*N*	*N*+	detected pesticide	mean ± SD	range	% area	% locations	*d* since sowing
F14	November	10	7	tebuconazole	19.27 ± 15.14	1.72–38.61	21.61	14.28	6
F18	November	9	3	tebuconazole	8.40 ± 2.93	6.08–11.70	18.65	33.3	5–15
F06	November	4	3	tebuconazole	14.16 ± 12.52	5.09–28.44	16.07	6.66	5
F15^b^	November	1	1	tebuconazole	2.83				
F26^b^	May	2	1	difenoconazole	22.10				

a *N* = number of analyzed individual samples and *N*+ = number of samples with detectable levels of pesticide residues. Average pesticide concentrations are provided for each positive flock [mean ± SD, and total range concentration, in ng/g wet weight (w. w.)]. Information on the spatial ecology of the flocks is also provided, including the percentage of the flock MCP area calculated for the last 72 h that overlapped with recently sown plots (% area), the percentage of the GPS locations recorded during the last 72 h that were on recently sown plots (% locations), and the minimum and maximum number of days elapsed from the sowing of those plots to the collection of dropping samples (d since sowing).

b Not associated with any GPS-tagged animal.

Previous studies conducted in central Spain have found tebuconazole residues in the digestive contents of 19.1% of the red-legged partridges hunted during the sowing season.^2,3^ A recent experimental study during which partridges were fed with wheat seeds treated with the recommended doses of tebuconazole for seed coating showed detection rates of this active ingredient after recent ingestion of 100% and 80% in digestive contents and feces, respectively.^14^ If this ratio between detectability in digestive contents and feces is applied to the fecal samples collected during the present study, we can estimate a 20.8% prevalence in the digestive contents of the sampled partridges, consistent with previously reported exposure levels.^2,3^

Outside the sowing season, pesticide detection in partridge feces was limited to a single case of difenoconazole in spring, probably related to a foliar application of crops with this fungicide. Difenoconazole-based products are approved for foliar use on the predominant crops in our study area (i.e., cereals and vineyards^32^). Nonetheless, the chances for birds to ingest pesticides used for foliar application are expected to be lower than for pesticides used for seed treatments,^33,34^ which explains the low incidence of pesticide detection outside the sowing season.

### Pesticide Residues Detected in Seeds Collected in Sown Fields

**Figure 1:**
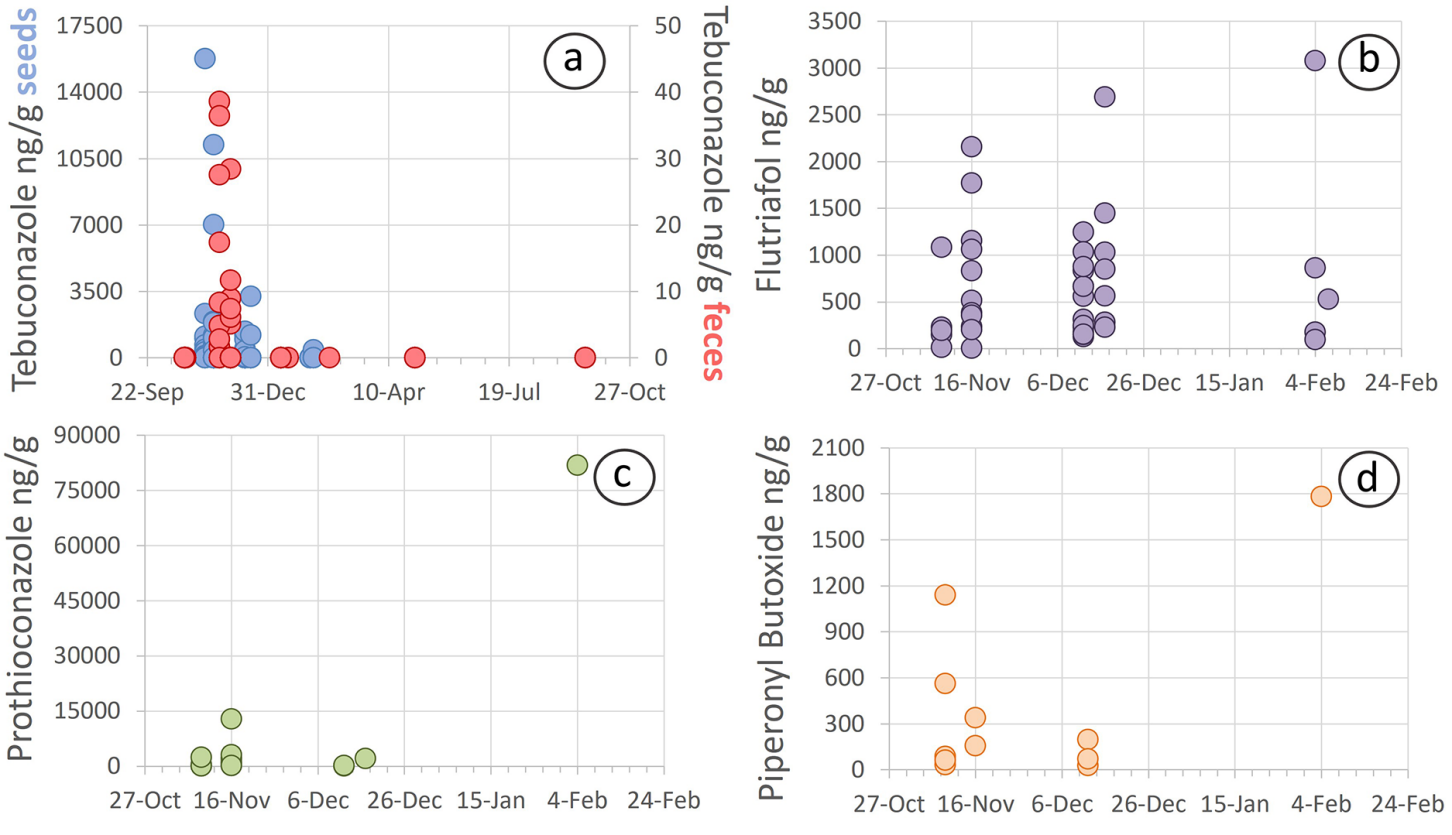
Dot plots showing concentrations of tebuconazole in feces (red) and seeds (blue) samples (a) and flutriafol (b), prothioconazole (c), and piperonyl butoxide (d) concentrations measured in seed samples collected over the course of the study.

Pesticides were detected in 83.2% of seed samples collected from sown fields (*n* = 101 plots), the majority of which corresponded to samplings in November and December (Figure 1). The detected products included the synergist piperonyl butoxide and the five triazole fungicides approved as seed treatments in the area, with tebuconazole (47.52%) and flutriafol (40.59%) as the most frequently detected products, followed by prothioconazole (20.79%), triticonazole (6.93%), and difenoconazole (0.99%). Flutriafol was the most detected active ingredient in barley seeds, and tebuconazole was the predominant product in oats and wheat seeds (Table S2). Most samples (*n* = 49; 48.51% of seed samples) contained a single active ingredient, but 29 samples (28.71%) contained two active ingredients, four samples (3.96%) contained three active ingredients, and two samples (1.98%) contained four active ingredients (Table S2).

Tebuconazole was the most frequently detected pesticide in partridge feces and seeds. Flutriafol and prothioconazole, despite their relatively high frequencies of use as seed treatments, were not detected in partridgeś feces. This could be explained by the different metabolisms of these compounds in birds.^14,35^ Flutriafol is quickly metabolized by partridges after its uptake as seed treatment, with a detection rate in feces that is only 28.6% immediately at the end of a 6 day period feeding exclusively on flutriafol-treated seeds. By contrast, the detection rate of one of its metabolites, 1,2,4-triazole, in the same fecal samples was 60%.^14^ This can explain why flutriafol, despite being used as seed treatment in our study area at a similar frequency as tebuconazole, was not detected in partridge feces and highlights the importance of considering the analysis of metabolites in fecal samples as a relevant addition to that of parent compounds for assessing exposure. Another possible explanation is that flutriafol-treated seeds could have a lower acceptance by partridges than tebuconazole-treated ones. Factors such as bird preference for some seeds, palatability of commercial formulations of plant protection products, or conditioned aversion effect due to the postingestion distress may influence the acceptance and consumption of certain pesticide-treated seeds by birds and, consequently, affect exposure to those pesticides.^4,36–38^ There is no published information on differential avoidance by birds of distinct triazole active ingredients or formulated products, but such differential avoidance has been observed experimentally with other types of pesticides.^37,38^

The detection of prothioconazole in seed samples was strongly linked to that of tebuconazole. In fact, 20 of the 21 seed samples that tested positive for prothioconazole also contained tebuconazole. According to the information provided by local farmers, most plots sown with these substances used the formulation Raxil Plus, which contains a mixture of the two active ingredients (25% prothioconazole and 15% tebuconazole). However, the co-occurrence of the two active ingredients was not observed in fecal samples, where only tebuconazole was detected. In addition, the prevalence of tebuconazole in seed samples (47.5%) was also higher than that of prothioconazole (20.8%). Detection rates of tebuconazole and prothioconazole in the feces of partridges experimentally fed with Raxil Plus-treated seeds during 6 days were 80 and 73%, respectively.^14^ This suggests a quicker metabolism of prothioconazole compared to tebuconazole, although the difference does not seem high enough to explain why the prevalence of tebuconazole was higher than that of prothioconazole in seeds and feces collected from the plots as part of the present study. The differences in prevalence between these active ingredients could be due to differences in environmental degradation, which is lower for tebuconazole than for prothioconazole (median degradation times—DT50—in soil are >1 year for tebuconazole and 0.07–1.27 days for prothioconazole^39,40^). We cannot exclude the possibility that farmers’ information was not complete, and other formulations were used in the study area, containing tebuconazole as the only active ingredient (e.g., Redigo). Although no prothioconazole-containing formulations other than Raxil Plus are approved for cereal seed treatment, one of the 21 seed samples that tested positive for this fungicide did not contain tebuconazole. This isolated case could come from a direct treatment made by a farmer that could have resulted in a nonhomogeneous coating of the seeds or that could have been made with a product not specifically approved for seed treatment.

### Relation of Pesticide Exposure to Diet

The determination of partridges’ diet was performed through a DNA-metabarcoding analysis of fecal samples to identify plants consumed by partridges. We focused on vegetal components only because adult red-legged partridges feed mostly on plants.^2,3,41^ Despite some controversy about the use of metabarcoding as a quantitative tool for diet composition,^41–44^ Portugal-Baranda et al.^45^ included mock samples and showed the usefulness for comparative diet studies of the same barcodes that we have used after finding an overall positive relationship between the real DNA abundance and the RRA obtained for five common plants (although, depending on the plant and the barcode used, the RRA could overestimate or underestimate the real abundance). Consequently, we have assumed that the RRA obtained from fecal samples would be a good proxy to quantify the different diet components of red-legged partridges.

The four PCs extracted from the PCA run on ITS2 gene results on partridge diet explained 87.8% of the variability of the six considered plant genera or groups, while the three PCs extracted from the analysis of rbcL data explained 73.9% of the variability (Table S3). For both genes, the first PC (PC1) was negatively associated with the consumption of barley and positively associated with the consumption of wild plants, the second PC (PC2) was negatively associated with the consumption of oats, and the third PC (PC3) was positively associated with pea consumption. In addition, wheat appeared negatively associated with the PC2 extracted from ITS2 analysis, and vetch was negatively associated with the PC4 of the ITS2 gene (Table S3). Contrary to expectations, none of the diet PCs explained fungicide detection in the feces collected from the same flock (ITS2: *X*^2^ = 0.828, 1 d.f., *p* = 0.363 and rbcL: *X*^2^ = 1.606, 1 d.f., *p* = 0.205).

**Figure 2:**
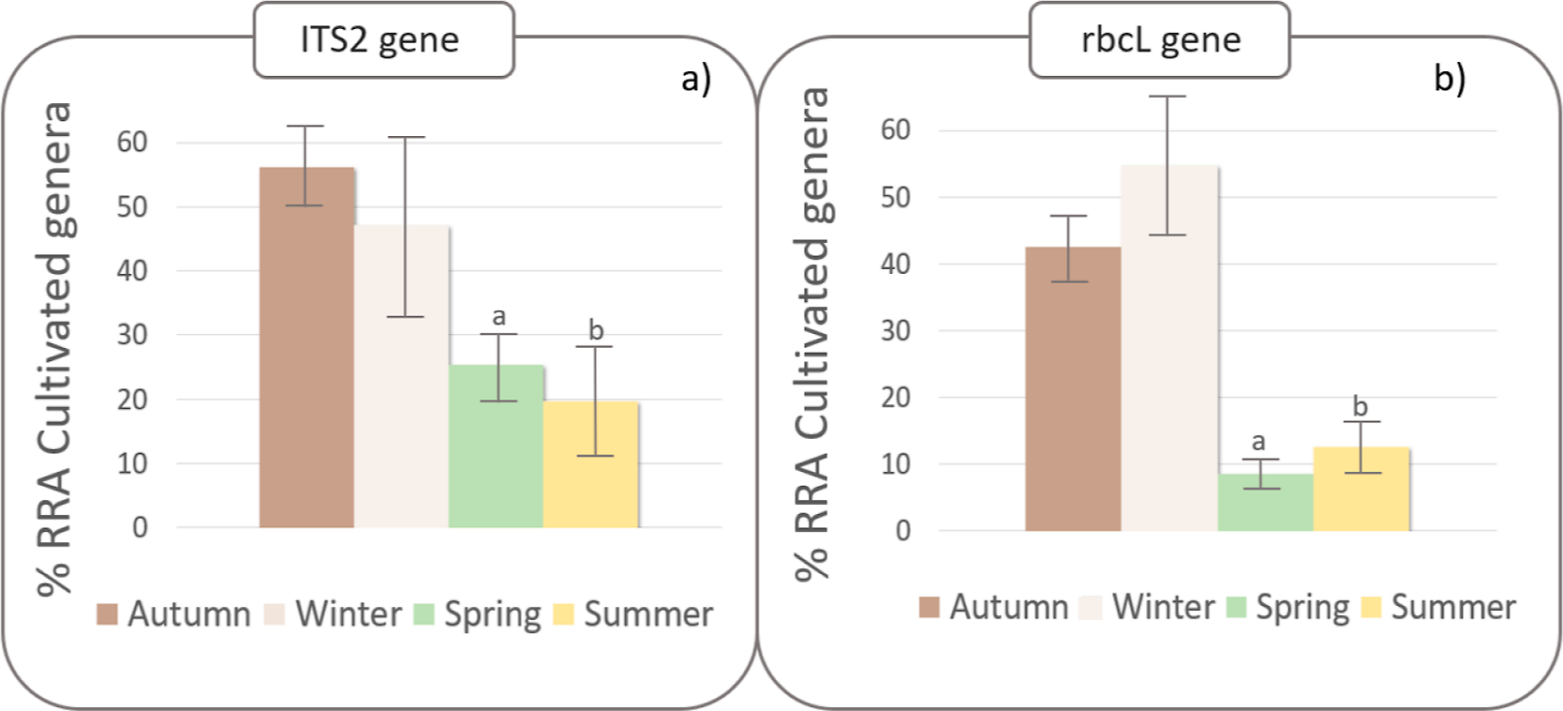
Relative read abundance (RRA %) with error bars ±SE of sequences corresponding to cultivated plant genera, resulting from the analysis of the two gene regions that were amplified for diet analysis: ITS2 (a) and rbcL (b). Different lower-case letters indicate significant (*p* < 0.05) differences between seasons according to least significant difference pairwise comparisons.

The diet analysis using both ITS2 (Wald’s *X*^2^ = 8.907, d.f. 3, *p* = 0.031) and rbcL (*X*^2^ = 32.677, d.f. = 3, *p* < 0.001) genes showed a greater consumption of cultivated plants by partridges during autumn and winter than in spring or summer (Table S4 and Figure 2). During autumn and winter, between 43.9 and 56.1% of the ingested products, depending on the used gene, corresponded to plant species of commonly cultivated genera. These percentages are consistent with results from crop content analyses,^2^ which revealed that sown seeds (i.e., cereal and legume seeds) accounted for 50.7% of the total biomass (fresh weight) ingested by red-legged partridges during the sowing season, and winter cereal seeds alone constituted 42.3% of that biomass. Lopez-Antia et al.^3^ also determined that, on average, 53.4% of the biomass in red-legged partridge digestive contents corresponded to winter cereal seeds, although that value was calculated from the analysis of crop contents of partridges hunted in seven Spanish provinces, among which a high variability was observed (from 26.5 to 89.3%). Barley was the most consumed genus during the sowing season (31.6–40.7%, depending on the gene used), which is consistent with its predominance among crops in the studied area (71.6% of the annual crop fields during the study season^32^).

Despite the evidence pointing to treated seeds as a major source for pesticide uptake by granivorous birds during the sowing season,^2,3,19,20,22^ the high dependence of partridge on cultivated plants and the widespread use of seed treatments in the study area, we did not find a statistical association between the consumption of cultivated plants (estimated from feces’ DNA analysis) and the detection of pesticides in fecal samples. This lack of association may be because partridges may have been feeding on cereals that were not treated with pesticides, like the left-over plants (i.e., grain and straw) from the previous harvest. Most experimental studies conducted to compare the consumption of treated and untreated seeds indicate that, when both choices are available, birds show a preference for untreated seeds.^36–38,46,47^ This pattern has also been observed in the field.^48,49^ Therefore, even if untreated seeds were less available than treated seeds in the study area, partridges might have selected, when possible, uncontaminated seeds, which could explain the lack of association between consumption of cultivated plants and exposure in partridges. Also, even if coming from treated seeds, the materials taken by partridges could have reduced pesticide loads either because of environmental degradation or because of pesticide dilution by plant growth,^50^ if the consumed materials are germinated seeds or shoots.^51^ Another possible reason that may influence the lack of association previously described between cultivated plant consumption and pesticide exposure in birds^2,3^ may be because the detection rate of these compounds in feces is not 100%,^14^ leading to potential false negatives in the data, which hinders the identification of this association. To elucidate the reasons behind the lack of correlation between cereal ingestion and pesticide presence in feces, further studies are needed to monitor how pesticide residues vary over time in seeds and shoots.

### Pesticide Exposure and Spatial Ecology

Pesticide (i.e., tebuconazole) exposure was confirmed for three out of the six partridge flocks for which we established an association between fecal samples and GPS tags, whereas no pesticide residues were detected in feces from the other three flocks. The GPS data analysis revealed that the MCP of the three exposed flocks overlapped with recently sown plots and that some of the recorded positions of these three flocks during the 72 h before feces collection were in recently sown plots (sown within the last 6 days; Table 1). By contrast, GPS data obtained from the three nonexposed flocks revealed no overlap with recently sown areas during the 72 h before feces collection (Figure S3). During autumn, pesticide detection in feces (i.e., pesticide exposure) was significantly higher in flocks that had recently visited sown plots (*p* = 0.008; Table S5).

**Figure 3:**
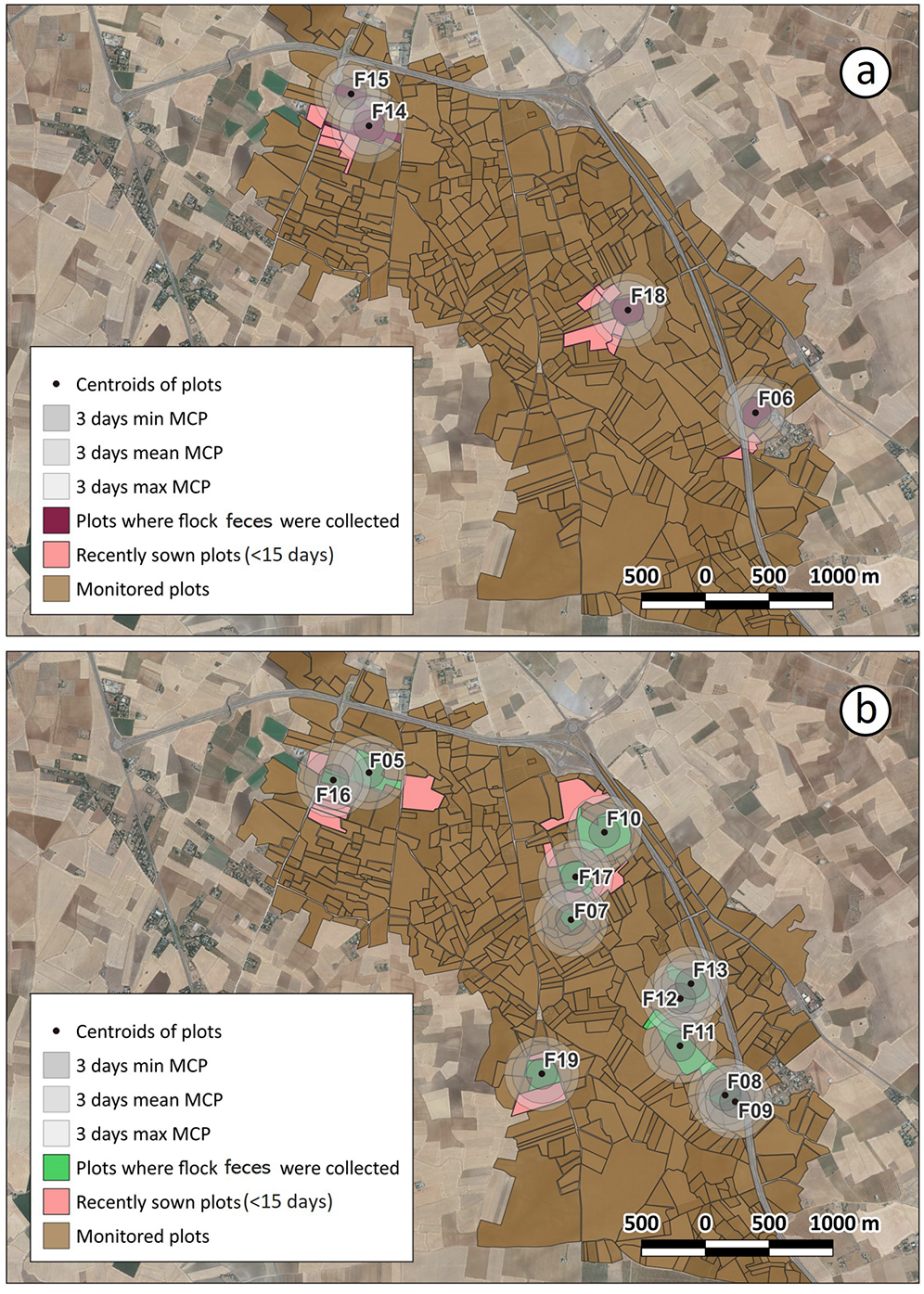
Spatial information for flocks (a) showing detectable pesticide residues in feces or (b) not showing detectable pesticide residues in feces. The figure shows the estimated circular home range areas around the centroids of plots from which partridge feces were collected, adjusted to the minimum, mean, and maximum surface areas of the estimated MCP of animals (see Figure S3). Note the overlap of circular areas (home ranges) with recent plot sowing (within the last 15 days). Letters and numbers refer to the flock IDs. Details on the % overlap with sown fields and on the time since the sowing day in each case are given in Table S6.

To establish an association between spatial ecology and pesticide exposure in those flocks without GPS-tracked birds, we inferred their home ranges during the days prior to sample collection by drawing circles of different radii around the feces collection sites. When the minimum MCP area was considered (i.e., radius = 121 m), we detected an overlap with a recently sown plot (sowing date ≤15 days) in 25% of samples with detectable pesticide levels and in 27.3% of samples with no pesticide detection (Table S5 and Figure 3). Using this radius, the detection of pesticides in partridge feces was not explained by the presence of recently sown plots in the minimum MCP area around the fecal collection site (*p* = 0.930; Table S5). When considering the mean MCP area (i.e., radius = 234 m), we detected a significant interaction (*p* < 0.001) between the tebuconazole detection in feces and the overlap of that flock with a recently sown plot (Table S5 and Figure 3). When we considered the maximum MCP area (i.e., radius = 296 m), the influence of visiting a recently sown plot on pesticide detection in feces was close to the defined threshold for statistical significance (*p* = 0.057, Table S5, and Figure 3).

The presence of recently sown plots, where seeds were expected to be available on the surface, within partridge home ranges was an important determinant of pesticide exposure. The need for assessing the ecotoxicological risk of pesticides using spatial-temporal data to predict exposure risk is increasingly being recognized.^5,20,21,52,53^ The spatial ecology and distribution patterns of farmland birds, including red-legged partridges, are influenced by agricultural practices, particularly cropland management and crop phenology (e.g., sowing and harvest).^15–17^ Previous research on the spatial ecology of red-legged partridges has shown that the occurrence of birds is related to the presence of wheat fields in agricultural areas, which are positively selected by partridges for feeding.^54,55^ This association would explain why the occurrence of birds in recently sown plots was a good predictor of pesticide residue detection in partridge feces. Therefore, gaining knowledge of birds’ spatial ecology associated with food availability can help us develop tools to estimate the risk of pesticide exposure through seed consumption.

### Implications for Environmental Risk Assessment

Our results suggest that the availability of sown seeds is a more sensitive indicator of partridge exposure to pesticides during the sowing season than the determination of diet from fecal DNA analysis. The study of diet and spatial ecology provided useful insights but with some limitations and uncertainty; on the one hand, even if DNA metabarcoding is a valid semiquantitative method to study diet composition, it does not differentiate between treated and pesticide-free materials of a given plant species. On the other hand, the analysis of home ranges (MCPs or concentric areas around the sites of fecal sample collection) was insufficient to determine if sown plots within these areas were used by partridges for feeding or not.

Another main and relevant source of uncertainty is the detectability of pesticide residues in feces. All fecal samples showing detectable residues of pesticides during autumn were collected in November, the month of the highest sowing activity. Samples collected during the other months with less intense sowing activity (October, December, and, to a lesser extent, January) tested negative for pesticides. This can be interpreted in two ways: on the one hand, the dependence of partridges on sowing seeds is maximum during the peak of sowing, but if uncontaminated food items are available, pesticide exposure may be reduced. This uncontaminated food was likely available at the beginning of autumn when old crops have not yet been plowed, and later in the season, when sown fields and weeds begin to germinate with the first autumn rains. Therefore, the peak of the sowing season in the middle of autumn (i.e., November during the study year) is not only when sown seed availability is highest but also when alternative food sources are scarce. On the other hand, feces were only useful to detect very recent ingestion of pesticides, as recently demonstrated by Fernández-Vizcaíno et al.^14^ Hence, the use of fecal sampling as a pesticide assessment method leads to an underestimation of the real exposure. Fecal sampling probably becomes a sensitive enough method only when there is low availability of uncontaminated food sources and partridges are more likely to feed repeatedly on treated seeds.

Although the exposure risk to pesticides from the consumption of treated seeds is concentrated within the sowing season, the consequences of that exposure for animals’ health and population viability can go beyond short-term effects after treated seed ingestion. The experimental exposure of partridges to triazole-treated seeds during winter at doses that resembled field situations resulted in long-term adverse effects on reproduction that manifested later in spring; those effects included reductions in fecundation rates,^26^ clutch and brood sizes,^11,12,56^ as well as alterations of egg-laying phenology.^12^ Likewise, field-based studies demonstrated that wild birds are susceptible to suffering from chronic toxicity after ingestion of triazole-treated seeds.^2,3^

Our results show that the likelihood of pesticide exposure is especially high during certain periods within the sowing season, which highlights the necessity of implementing measures to mitigate exposure risk during those critical periods. Crop management and modified sowing techniques could contribute to reducing surface seed availability and exposure, for instance, by increasing sowing depth and using a roller after sowing to maximize the proportion of properly buried seeds.^57,58^ In addition, increasing areas with natural vegetation, such as field margins, pastures, or fallows, in agricultural environments can be promoted to increase alternative food availability for farmland birds.^59–61^ In this context, Fernández-Vizcaíno et al.^2^ showed that fungicide uptake by partridges was reduced in landscapes with a higher heterogeneity, where the availability of natural vegetation was higher compared to landscapes with a high surface area occupied by crop fields. Our results highlight the importance of prioritizing the implementation of those risk mitigation measures during the peak of the sowing season when partridges are more likely to exploit pesticide-treated seeds as a food resource.

## Supporting Information Available

Pesticide residue analysis and DNA-metabarcoding techniques, ESI-MS parameters used for pesticide analyses, occurrence of pesticide-treated seeds in monitored sown plots, results of the PCA conducted on the diet variables for ITS2 and rbcL genes, mean RRA of cultivable genera and wild plants for ITS2 and rbcL genes, contingency tables to relate pesticide detection in feces, data of overlap area (%) between recently sown plots and circular home ranges of different diameters, showing the minimum and maximum number of days elapsed between sowing of those plots and the day of sample collection, study area showing the main land use, schematic representation of the study design throughout the different seasons, and a map showing GPS locations and corresponding MCP of wild red-legged partridges (PDF)
