# Supplementary material for: Diet and Spatial Ecology Influence Red-Legged Partridge Exposure to Pesticides Used as Seed Treatment
Source: Environ Sci Technol. 2023 Sep 25;57(40):14861–70. doi: 10.1021/acs.est.3c03905 (PMC10569034; doi:10.1021/acs.est.3c03905)
Supplement: Supplementary file 1 — es3c03905_si_001.pdf [file es3c03905_si_001.pdf]

Supporting Information

Diet and spatial ecology influence red-legged partridge exposure to pesticides used as seed treatment

Elena Fernández-Vizcaíno[1], François Mougeot[1], Xabier Cabodevilla[2,3], Mario Fernández-Tizón[1], Rafael Mateo[1], María J. Madeira[4] and Manuel E. Ortiz-Santaliestra[1]

[1] Instituto de Investigación en Recursos Cinegéticos (IREC) CSIC-UCLM-JCCM, Ronda de Toledo 12, 13005 Ciudad Real, Spain.

[2] Conservation Biology Group, Landscape Dynamics and Biodiversity Program, Forest Science and Technology Centre of Catalonia (CTFC), km 2 25280 Solsona, Spain

[3] Terrestrial Ecology Group (TEG-UAM), Department of Ecology, Universidad Autónoma de Madrid, Calle Darwin 2, 28049 Madrid, Spain

[4] Department of Zoology and Animal Cell Biology, Faculty of Pharmacy, University of the Basque Country (UPV/EHU), Paseo de la Universidad 7, 01006 Vitoria-Gasteiz, Alava, Spain

*E-mail contact: efvval@hotmail.com

Number of pages: 12

Number of tables: 6

Number of figures: 3

Supporting Information

1. MATERIALS AND METHODS......3
1.1. Pesticide residue analysis......3
1.2. Diet......3
2. TABLES......5
Table S1......5
Table S2......5
Table S3......6
Table S4......7
Table S5......8
Table S6......9
3. FIGURES......10
Figure S1......10
Figure S2......10
Figure S3......11
4. REFERENCES......12

1. MATERIALS AND METHODS

1.1. Pesticide residue analysis

For pesticide residue analysis in faeces and seeds, we weighed about 0.3 g of sample to which we added 50 µl of aldicarb at 20 ng/µl as the internal standard. Samples were extracted with 3 ml of acetonitrile by 5 min of vortex followed by sonication during another 5 min. Finally, 1 ml of extract was filtered through a 0.2 µm nylon syringe filter and transferred to a glass vial for chromatography. We used a Zorbax Eclipse XBD-C18 (4.6 x 150 mm, 5 µm) chromatographic column with a Zorbax Eclipse XBD-C18 (4.6 x 10 mm 5 µm) precolumn. The injection volume was 20 µL. The chromatographic conditions consisted of an elution gradient of two mobile phases (A: 0.01% formic acid in milliQ water; B: 0.01% formic acid in acetonitrile). The initial conditions were 95% of A and 5% of B, reaching 50% of A and 50 of % B at min 8.5, and 10% of A and 90% of B at 25 min. This was maintained until min 28, returning to the initial conditions by min 29. The flow rate was 0.8 mL/min. Pesticides were detected using positive ions monitored with the following settings: nebulizer pressure 35 psi, drying gas flow 8 $l \cdot min^{-1}$, drying gas temperature 250 °C, vaporizer temperature 200 °C, capillary voltage 3500 V, and charging voltage 1000 V. The monitored ions for each pesticide along with the retention time and the fragmentation voltage for each ion are shown in Table S1. Pesticide standards were obtained from Dr. Ehrenstorfer (Augsburg, Germany) and Supelco (Merck, Darmstadt, Germany). Concentrations for calibration curves ranged from 0.1 to 0.5 µg/ml in acetonitrile. The inter-day recoveries (±SD) achieved in our laboratory using this method ranged from 78.8 ± 5.4% to 106.2 ± 12.4% [1,2], being in accordance with the SANTE/12.682/2019 guidelines of the European Union [3]. These values were calculated based on fortified control samples of farmed partridge faeces analysed during 5 consecutive days.

1.2. Diet

To describe the plant diet of red-legged partridges, we applied DNA-metabarcoding techniques to faecal samples, a technique that has proven to be very useful for conducting diet studies [4,5], providing relevant information that was previously not possible with traditional techniques. Although the DNA extraction was carried out on each individual sample, the flock (group of birds roosting together) was used as the study unit (n=32 flocks). Faeces were analysed as described by Portugal-Baranda et al. [6], DNA extractions were performed using the QIAamp® DNA Stool Mini Kit from Quiagen (Ref. 51504). For each DNA sample pool (flock), two DNA regions were amplified by PCR at the Analytical Services

(SGIker) of the University of the Basque Country (UPV/EHU). The internal transcribed spacer 2 (ITS2) region of nuclear ribosomal DNA was amplified using the primers 5′-TGTGAATTGCARRATYCMG-3′ (forward) and 3′-CCCGHYTGAYYTGRGGTCDC-5′ (reverse), and the large-chain subunit of the ribulose-1,5-bisphosphate carboxylase/oxygenase (rbcL) region was amplified using the primers 5′-CTTACCAGYCTTGATCGTTACAAAGG-3′ (forward) and 3′-GTAAAATCAAGTCCACCRCG-5′ (reverse), following the protocols described by Erickson et al.[7] and Moorhouse-Gann et al. [8], respectively. The amplification was carried out using the same DNA quantity, reaction mix and thermocycler conditions as described by Portugal-Baranda et al. [6]. Samples were purified and a second reaction was performed to index each amplified product and attach Illumina adaptors using the Illumina Nextera v2 kit. PCR outputs were sequenced in an Illumina MiSeq NGS platform (sequencing of 2 × 250 bp paired-end reads) with the MiSeq Reagent Kit v2, following the manufacturer's instructions. We included blanks within the library construction; these were sequenced and we did not reveal any contamination, neither in the PCR products nor in the sequencing output.

Once the sequences were obtained, we proceeded to bioinformatic analysis, as described by Portugal-Baranda et al. [6], using the Cutadapt software (Martin 2011) and the DADA2 v1 package[9] in R v4.1.2 computer software (R Core Team, 2021). First the primers were removed from the sequencing output using the Cutadapt software (Martin 2011). Then, using the *filterAndTrim* function, the reads were filtered based on their quality, and trimmed to 210 bp in the case of forward sequence and to 200 bp in the case of reverse sequences. Subsequently, these forward and reverse sequences were merged (*mergePairs*), we built a table of Amplicon Sequence Variants (ASVs) (*makeSequenceTable*), and the chimeric sequences were removed (*removeBimeraDenovo*). Finally, the taxonomy was assigned (*assignTaxonomy*) using two reference databases, one per barcode, and a minBoot of 80. This implies that during the taxonomic assignment, 100 classification simulations were run, and a given taxon was considered valid when it was assigned in at least 80% of the simulations. The construction of the reference database was based on that provided by Moorhouse-Gann et al. [8] for ITS2, which included 1913 unique sequences, and on that provided by Bell et al. [10] for rbcL, which included 18779 sequences that were subsequently completed with sequences obtained by Sanger sequencing from regional plant species and some additional sequences obtained from Genbank® (National Center for Biotechnology Information, NCBI) (see Portugal-Baranda et al., [6]).

All the ASVs that did not represent at least 0.01% of the reads of at least one flock were removed from the analysis to calculate the relative read abundance (RRA). Although some ASVs could be identified at the species level, all ASVs were finally unified at the genus level, so the plant genus was our unit of analysis for the study of the partridges' diet. Finally, each plant genus was classified as

"crop" (if it included cultivated species that are commonly grown in the study region, i.e. barley, wheat, oats, pea and vetch) or else as "wild plants".

2. TABLES

Table S1. ESI-MS parameters used for pesticide analyses. RT: retention time in minutes.

Chemical	RT	Polarity	Monitored ions				LOD[c] (ng/g)	LQ[d] (ng/g)
			Quantifier (V)	Qualifiers (V)				
Carbendazim†	2.39	Positive	160(150)	192(125)	134(125)		14.78	49.27
Thiamethoxam[b,*]	7.05	Positive	292(75)	211(125)	248(125)	181(150)	3.12	10.40
Clothianidin[b,*]	8.45	Positive	250(75)	132(125)			1.43	4.77
Imidacloprid[b,*]	8.79	Positive	256 (100)	209 (200)	257 (100)	211 (150)	3.53	11.79
Thiacloprid[b]	10.20	Positive	253(120)	126(160)	255(120)	128 (160)	4.80	16.02
Paraquat†	11.50	Positive	304(200)	305(200)	117(300)	147(250)	93.39	311.32
Flutriafol[a]	12.28	Positive	302 (150)	303 (100)	70 (200)	74 (300)	6.92	23.08
Thiram[a,*]	12.9	Positive	88 (100)	303 (100)	73 (100)		1.82	6.09
Metalaxyl-M[a]	13.00	Positive	280 (50)	220 (150)	160 (200)	221 (150)	33.13	110.44
Triticonazole[a]	15.00	Positive	318 (150)	320 (100)	319 (150)	288 (150)	5.90	19.67
Tebuconazole[a]	16.80	Positive	308 (150)	70 (200)	309 (150)	288 (150)	8.39	27.97
Prothioconazole[a]	17.80	Positive	326(150)	344(100)	328(150)	346(100)	2.96	9.88
Fluoxastrobin[b]	17.97	Positive	427(200)	459(150)	429(200)	461(125)	17.49	58.32
Difenoconazole[a]	19.05	Positive	406 (100)	408 (100)			6.68	22.29
Pyraclostrobin[b]	20.68	Positive	388(100)	194(125)	163(150)	105(200)	7.14	23.82
Piperonyl Butoxide[a]	24.20	Positive	177 (150)	356 (100)	147 (200)	361 (200)	3.58	11.93

[a] Active ingredients included in formulations authorized for use as cereal seed treatment in Spain during the study period (MAPA, 2021).
[b] Active ingredients included in formulations authorized as Plant Protection Products in Spain during the study period, but not for cereal seed treatment (MAPA, 2021).
†Chemicals that were not authorized as plant protection products in the European Union during the study period (https://ec.europa.eu/food/plant/pesticides/eu-pesticides-database/active-substances/)
*Chemicals that have been discontinued from the list of approved plant protection products in the European Union after the study took place (https://ec.europa.eu/food/plant/pesticides/eu-pesticides-database/active-substances/)
[c] Limit of detection (LOD) calculated from the standard deviation of the lowest point of the calibration curve multiplied by 3.
[d] Limit of quantification (LQ) calculated from the standard deviation of the lowest point of the calibration curve multiplied by 10.

Table S2. Occurrence of pesticide treated seeds in monitored sown plots (n=101). Results are given by crop type for each of the active ingredients or combinations of these. Some of the monitored plots could not be assigned a specific crop type because more than one seed type was observed in the field surface.

		All plots (N=101)	Barley (N=72)	Oats (N=14)	Wheat (N=6)	Pea (N=2)
Active ingredients	Difenoconazole	0.99	0.00	0.00	16.67	0.00
	Flutriafol	40.59	48.61	7.14	33.33	50.00
	Prothioconazole	20.79	22.22	0.00	33.33	0.00
	Tebuconazole	47.52	45.83	50.00	66.67	50.00
	Triticonazole	6.93	2.78	35.71	0.00	0.00
	Piperonyl butoxide	9.90	9.72	14.29	16.67	0.00
Number of products	1 fungicide	47.52	54.17	21.43	33.33	100.00
	2 fungicides	23.76	22.22	21.43	33.33	0.00
	3 fungicides	1.98	1.39	7.14	0.00	0.00
	1 fungicide + synergist	4.95	5.56	7.14	0.00	0.00
	2 fungicides + synergist	1.98	1.39	0.00	16.67	0.00
	3 fungicides + synergist	1.98	2.78	0.00	0.00	0.00

Table S3. Results of the Principal Component Analysis conducted on the diet variables for ITS2 and rbcL genes. Correlation coefficients between plant groups and PCs, as well as individual and cumulative explained variance and eigenvalues of each PC, are shown.

	ITS2				rbcL		
	Diet PC1	Diet PC2	Diet PC3	Diet PC4	Diet PC1	Diet PC2	Diet PC3
Barley	**-0.987**	0.006	-0.086	-0.092	**-0.848**	0.358	0.206
Oats	0.305	**-0.749**	-0.160	0.506	0.035	**-0.940**	-0.262
Wheat	-0.129	**0.691**	-0.115	0.523	0.362	0.460	-0.530
Pea	0.127	-0.035	**0.891**	-0.238	0.358	-0.089	**0.797**
Vetch	0.385	0.050	-0.540	**-0.629**	-0.547	0.093	-0.127
Wild	**0.838**	0.368	0.052	0.113	**0.890**	0.285	-0.023
Variance							
Proportion	32.500	19.638	18.920	16.715	34.502	22.021	17.410
Cumulative	32.500	52.138	71.058	87.773	34.502	56.523	73.933
Eigenvalue	1.950	1.178	1.135	1.003	2.070	1.321	1.045

Table S4. Mean relative read abundance SD (and range in brackets) of cultivable genera and wild plants for ITS2 and rbcL genes at different times of the year consumed by red-legged partridge flocks.

	ITS2							rbcL						
	N (flocks)	barley	oats	wheat	pea	vetch	wild plants	N (flocks)	barley	oats	wheat	pea	vetch	wild plants
Autumn	15	40.71±28.48 (5.15-99.31)	7.93±13.82 (0.01-50.74)	0.59±2.24 (0.00-8.70)	3.82± 9.75 (0.00-29.08)	3.07± 9.25 (0.00-35.75)	43.89±23.11 (0.67-86.76)	14	32.29±22.19 (7.12-75.66)	8.45±16.05 (0.00-60.12)	0.24±0.82 (0.00-3.07)	0.01±0.03 (0.00-0.11)	1.61±3.77 (0.00-12.13)	57.40±22.51 (15.96-86.27)
Winter	5	31.60±32.82 (4.11-85.46)	10.50±8.34 (2.08-19.64)	0.12±0.25 (0.00-0.57)	0.01±0.01 (0.00-0.03)	4.95±7.11 (0.00-15.66)	52.82±32.71 (9.74-91.51)	5	46.09±31.63 (10.65-84.96)	5.42±4.26 (0.72-10.16)	0.01±0.02 (0.00-0.05)	0.00±0.00 (0.00-0.00)	3.20±4.43 (0.00-9.19)	45.28±31.98 (7.10-88.56)
Spring	8	14.57±16.36 (0.03-41.79)	6.39±8.81 (0.01-21.35)	1.60±3.73 (0.00-10.63)	0.01±0.01 (0.00-0.04)	2.73±6.47 (0.00-18.44)	74.69±22.41 (49.66-99.56)	8	3.40±2.17 (0.08-6.57)	3.74±4.79 (0.00-12.41)	0.24±0.52 (0.00-1.50)	0.00±0.00 (0.00-0.00)	1.24±3.22 (0.00-9.21)	91.38±7.07 (77.87-99.58)
Summer	4	16.04±12.54 (0.25-30.94)	2.57±5.13 (0.00-10.27)	0.61±1.23 (0.00-2.46)	0.45±0.90 (0.00-1.79)	0.00±0.00 (0.00-0.01)	80.33±17.39 (56.33-97.96)	3	12.54±10.25 (0.71-18.46)	0.00±0.00 (0.00-0.00)	0.00±0.00 (0.00-0.00)	0.00±0.00 (0.00-0.00)	0.00±0.00 (0.00-0.01)	87.45±10.25 (81.54-99.29)

Table S5. Contingency tables to relate pesticide detection in faeces to the use of recently sown fields considering the different approaches described for the spatial ecology study: data from the 6 GPS-monitored flocks that could be linked with a given faecal sample, and data from the 15 sampled flocks in autumn considering circular areas around the site of faeces collections with an extension equal to the minimum, mean or maximum MCP calculated for partridges. Data on each cell indicate the number of flocks, and percentages between parenthesis are calculated relative to each row total. Results of the chi-square test to compare frequencies among table cells are shown.

		GPS-tracked flocks			All flocks (minimum area extension)			All flocks (mean area extension)			All flocks (maximum area extension)		
		Overlap with sown fields	No overlap with sown fields	Total	Overlap with sown fields	No overlap with sown fields	Total	Overlap with sown fields	No overlap with sown fields	Total	Overlap with sown fields	No overlap with sown fields	Total
Pesticides detected in faeces		3 (100%)	0 (0%)	3	1 (25%)	3 (75%)	4	4 (100%)	0 (0%)	4	4 (100%)	0 (0%)	4
Pesticides not detected in faeces		0 (0%)	3 (100%)	3	3 (27.3%)	8 (72.7%)	11	3 (27.3%)	8 (72.7%)	11	5 (45.5%)	6 (54.5%)	11
Total		3 (50%)	3 (50%)	6	4 (23.7%)	11 (73.3%)	15	5 (33.3%)	10 (66.7%)	15	9 (60%)	6 (40%)	15
Chi-square test	X^2	7.00			0.008			10.909			3.636		
	d.f.	1			1			1			1		
	p	0.008			0.930			<0.001			0.057		

Table S6. Overlap (%) between recently sown plots and circular home ranges of different diameters around the sample collection site of study partridge flocks (areas correspond to minimum, mean and maximum MCP areas used by birds for 3 days). The minimum and maximum number of days elapsed between sowing of those plots and the day of sample collection (d since sowing) are also indicated for each sampled flock. Flock data are regrouped showing first flocks with pesticide detection (exposed) or without detection (non-exposed).

	Flock	Minimum area		Mean area		Maximum area	
	ID	% overlap	d since sowing	% overlap	d since sowing	% overlap	d since sowing
Exposed flocks	F14	0	-	11.30	7-11	14.27	7-11
	F15	0	-	0.61	11	3.09	11
	F18	0.75	6	7.03	6	9.39	6
	F06	0	-	2.68	5	1.72	5-8
Non-exposed flocks	F05	0	-	0	-	0.46	0
	F07	0	-	0	-	0	-
	F08	0	-	0	-	0	-
	F09	0	-	0	-	0	-
	F10	0	-	0	-	6.97	0
	F11	0	-	0	-	0	-
	F12	0	-	0	-	0	-
	F13	0	-	0	-	0	-
	F16	39.73	6-11	55.58	6-11	46.07	6-11
	F17	12.87	0-13	20.16	0-13	56.87	0-13
	F19	7.36	9-13	32.28	9-13	27.42	9-13

3. FIGURES

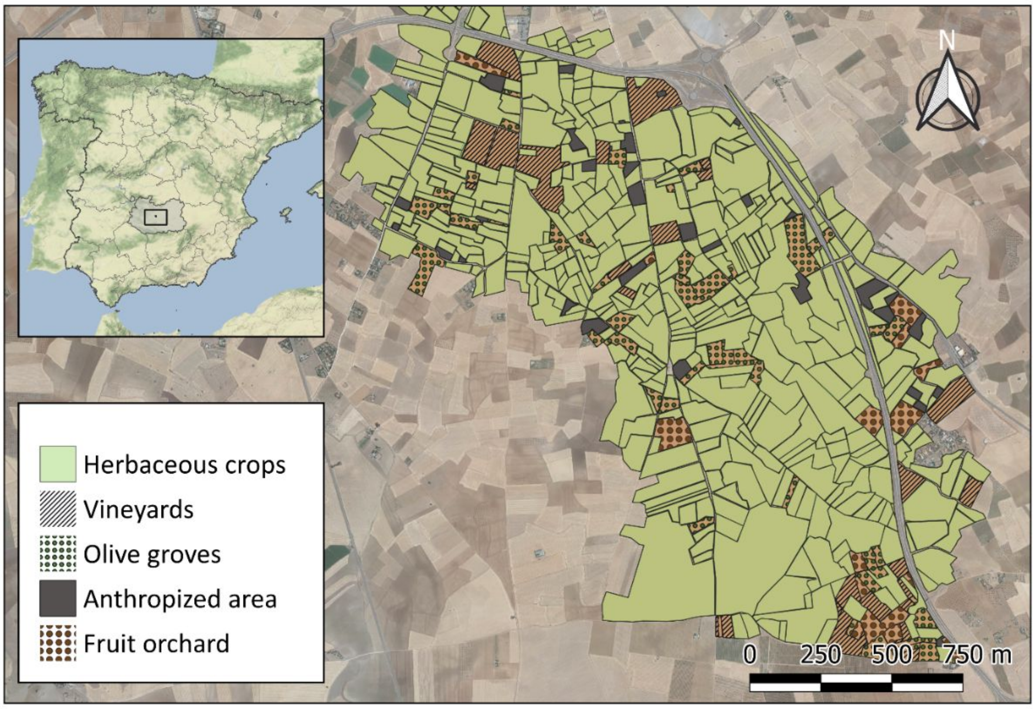

Figure S1. Location of the study area in central Spain and map showing the main land uses.

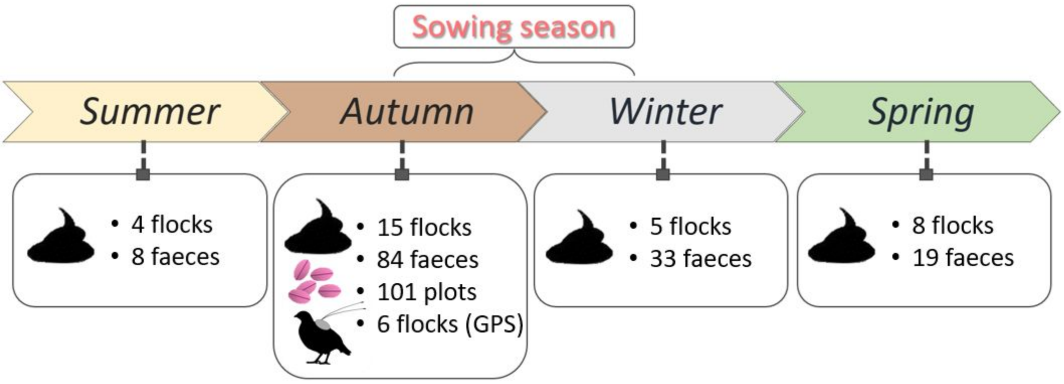

Figure S2. Schematic representation of the study design throughout the different seasons, indicating the seasons of sown seed and faeces collections and of recording of partridge location data for the spatial ecology study.

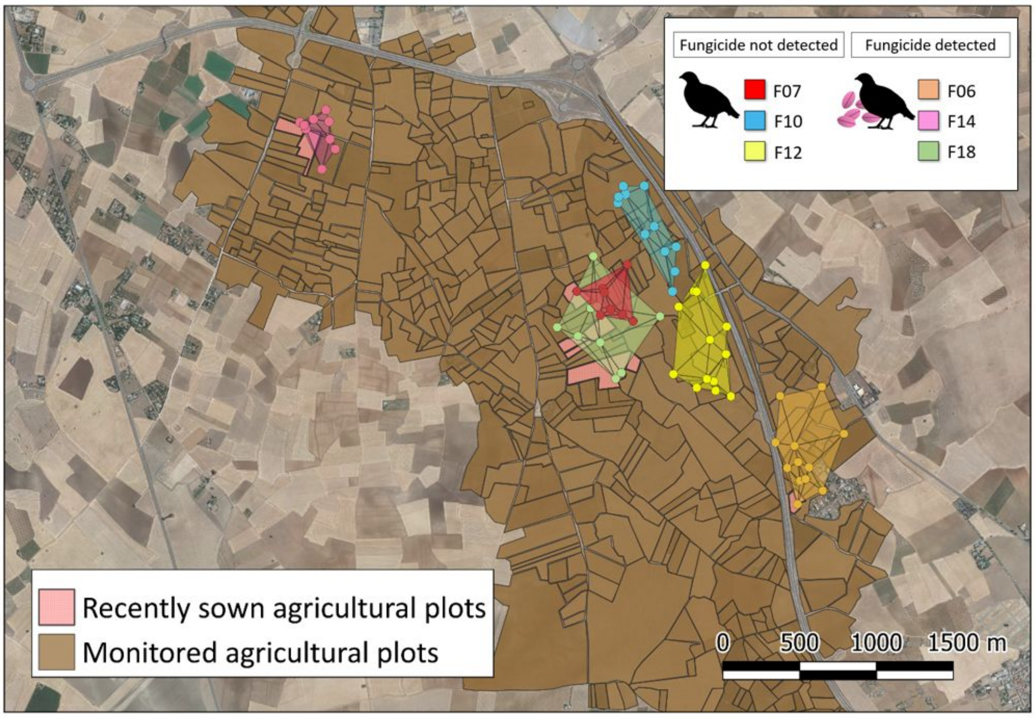

Figure S3. GPS locations and corresponding minimum convex polygons (MCP) of wild red-legged partridges from which faeces were collected. MCPs were calculated using bird locations during the 3 days before faeces collection. Note that MCPs overlapped with recently sown plots when fungicide exposure was detected (flocks F06, F14 and F18) but not in other flocks (F07, F10 and F12). The patch indicated as sown within the MCP corresponding to flock F07 was sown after the collection of faeces from that flock.

4. REFERENCES

(1) Lopez-Antia, A.; Ortiz-Santaliestra, M. E.; Mougeot, F.; Mateo, R. Experimental Exposure of Red-Legged Partridges (Alectoris Rufa) to Seeds Coated with Imidacloprid, Thiram and Difenoconazole. *Ecotoxicology* **2013**. https://doi.org/10.1007/s10646-012-1009-x.

(2) Fernández-Vizcaíno, E.; Mougeot, F.; Mateo, R.; Camarero, P. R.; Alcaide, V.; Ortiz-Santaliestra, M. E. A Non-Invasive Method to Monitor Farmland Bird Exposure to Triazole Fungicides. *Chemosphere* **2023**, *325*, 138316. https://doi.org/10.1016/j.chemosphere.2023.138316.

(3) European Comission. *Guidance Document on Analytical Quality Control and Method Validation Procedures for Pesticides Residues Analysis in Food and Feed, SANTE/12682/2019*; 2019.

(4) Pompanon, F.; Deagle, B. E.; Symondson, W. O. C.; Brown, D. S.; Jarman, S. N.; Taberlet, P. Who Is Eating What: Diet Assessment Using next Generation Sequencing. *Mol Ecol* **2012**, *21* (8), 1931–1950. https://doi.org/10.1111/j.1365-294X.2011.05403.x.

(5) Cabodevilla, X.; Mougeot, F.; Bota, G.; Mañosa, S.; Cuscó, F.; Martínez-García, J.; Arroyo, B.; Madeira, M. J. Metabarcoding Insights into the Diet and Trophic Diversity of

Six Declining Farmland Birds. *Sci Rep* **2021**. https://doi.org/10.1038/s41598-021-00519-9.

(6) Portugal-Baranda, T.; Mougeot, F.; Ortiz-Santaliestra, M. E.; Madeira, M.; Fernández-Vizcaíno, E.; Cabodevilla, X. Metabarcoding Reveals Seasonal Variations in the Consumption of Crops and Weeds by Wild Red-Legged Partridges. *Agric Ecosyst Environ*.

(7) Erickson, D. L.; Reed, E.; Ramachandran, P.; Bourg, N. A.; McShea, W. J.; Ottesen, A. Reconstructing a Herbivore's Diet Using a Novel RbcL DNA Mini-Barcode for Plants. *AoB PLANTS*. 2017. https://doi.org/10.1093/aobpla/plx015.

(8) Moorhouse-Gann, R. J.; Dunn, J. C.; De Vere, N.; Goder, M.; Cole, N.; Hipperson, H.; Symondson, W. O. C. New Universal ITS2 Primers for High-Resolution Herbivory Analyses Using DNA Metabarcoding in Both Tropical and Temperate Zones. *Sci Rep* **2018**. https://doi.org/10.1038/s41598-018-26648-2.

(9) Callahan, B. J.; McMurdie, P. J.; Rosen, M. J.; Han, A. W.; Johnson, A. J. A.; Holmes, S. P. DADA2: High-Resolution Sample Inference from Illumina Amplicon Data. *Nat Methods* **2016**. https://doi.org/10.1038/nmeth.3869.

(10) Bell, K. L.; Batchelor, K. L.; Bradford, M.; McKeown, A.; MacDonald, S. L.; Westcott, D. Optimisation of a Pollen DNA Metabarcoding Method for Diet Analysis of Flying-Foxes (Pteropus Spp.). *Aust J Zool* **2021**. https://doi.org/10.1071/ZO20085.
